# Dysfunction in endocannabinoids, palmitoylethanolamide, and degradation of tryptophan into kynurenine in individuals with depressive symptoms

**DOI:** 10.1186/s12916-024-03248-8

**Published:** 2024-01-25

**Authors:** Stefano Comai, Nicolas Nunez, Tobias Atkin, Maykel F. Ghabrash, Rita Zakarian, Allan Fielding, Marie Saint-Laurent, Nancy Low, Garrett Sauber, Eugenio Ragazzi, Cecilia J. Hillard, Gabriella Gobbi

**Affiliations:** 1 Department of Pharmaceutical and Pharmacological Sciences, University of Padua, Padua, Italy; 2 Department of Biomedical Sciences, University of Padua, Padua, Italy; 3 Department of Psychiatry, McGill University, Montreal, QC Canada; 4 IRCCS San Raffaele Scientific Institute, Milan, Italy; 5 Department of Psychiatry, McGill University Health Center, Montreal, QC Canada; 6 Neuroscience Research Center and Department of Pharmacology and Toxicology, Medical College of Wisconsin, Milwaukee, WI USA

**Keywords:** Depression, Endocannabinoids, Biomarkers, Serotonin, Kynurenine pathway

## Abstract

**Background:**

The endocannabinoid (eCB) system and the serotonin (5-HT) are both implicated in the severity of the depression. 5-HT is synthesized from the amino acid tryptophan (Trp), which is also a precursor for kynurenine (Kyn) whose production is increased at the expense of 5-HT in depressed patients. No clinical studies have investigated the crosstalk between the eCB system and the Trp/5-HT/Kyn pathways. Here, we hypothesized that the eCB system is associated with an enhanced Kyn production in relation to the severity of depressive symptoms.

**Methods:**

Eighty-two subjects (51 patients with a diagnosis of depressive disorder (DSM-5) and 31 healthy volunteers), were assessed with the Montgomery-Åsberg Depression Rating Scale (MADRS), Beck Depression Scale, and Global Clinical Impression. Serum concentrations of eCBs (*N*-arachidonoylethanolamine (AEA) and 2-arachidonoylglycerol (2-AG)); structurally related fatty acyl compounds 2-oleoylglycerol (2-OG), oleoylethanolamide (OEA), and palmitoylethanolamide (PEA); Trp, Kyn, Kyn/Trp ratio (an index of Trp degradation into Kyn) and 5-HT were also determined.

**Results:**

Following a principal component analysis including the severity of depression, Kyn and the Kyn/Trp ratio appear to be directly associated with 2-AG, AEA, and PEA. Interestingly, these biomarkers also permitted to distinguish the population into two main clusters: one of individuals having mild/severe depressive symptoms and the other with an absence of depressive symptoms. Using parametric analysis, higher serum levels of 2-AG, Kyn, and the ratio Kyn/Trp and lower levels of Trp and 5-HT were found in individuals with mild/severe depressive symptoms than in those without depressive symptoms. While in asymptomatic people, PEA was directly associated to Trp, and OEA indirectly linked to 5-HT, in individuals with depressive symptoms, these correlations were lost, and instead, positive correlations between AEA and 2-AG, PEA and AEA, and PEA vs 2-AG and OEA concentrations were found.

**Conclusions:**

Parametric and non-parametric analyses suggest a possible association between eCBs, tryptophan/kynurenine biomarkers, and severity of depression, confirming a likely interplay among inflammation, stress, and depression. The enhanced relationships among the biomarkers of the 2-AG and AEA pathways and related lipids seen in individuals with depressive symptoms, but not in asymptomatics, suggest an altered metabolism of the eCB system in depression.

**Supplementary Information:**

The online version contains supplementary material available at 10.1186/s12916-024-03248-8.

## Background

Major depressive disorder (MDD) affects about 5% of the population in industrialized countries [1]. MDD is one of the leading contributors to disability worldwide having significant implications for the quality of life of affected individuals and their families and being associated with elevated health care costs [2, 3]. Current treatments are fully effective in only one-third of treated patients, while another one-third show no response at all [4, 5]. Additionally, these treatments can produce several adverse effects that impact treatment adherence. Novel “fast-acting” antidepressants like ketamine have been approved for treatment resistant depression [6], but the limited time window of effectiveness (2–3 weeks) and the absence of long-term data on the safety and effectiveness of repeated dosing remain significant challenges [7]. Nevertheless, while ad hoc studies are still necessary, viewed from a clinical standpoint, there appears to be promise in maintenance treatment involving ketamine, as highlighted in a recent review by Smith-Apeldoorn et al. [8]. The therapeutic limitations of current antidepressants reflect a general lack of knowledge of the pathophysiological mechanisms of depression [9]. Thus, more research is needed to better understand the neurobiological underpinnings of depression and to discover and validate novel potential targets for drug development.

Multiple and independent lines of evidence derived from both preclinical and clinical studies suggest that the metabolism of the essential amino acid tryptophan (Trp) and the endocannabinoid (eCB) system contribute to the pathophysiology of MDD. Trp is the precursor of both serotonin (5-HT) and kynurenine (Kyn) through the so-called Kyn pathway. In this pathway, Trp is converted to Kyn by both the indoleamine 2,3-dioxygenase (IDO) enzyme that is activated by inflammation and the immune response [10] and the liver enzyme, tryptophan 2,3-dioxygenase (TDO), which is activated by Trp itself and the stress hormone cortisol [10]. In conditions of stress, mood disorders [11, 12], and suicidal behavior [13–17], the shunting of Trp from the synthesis of 5-HT to the Kyn pathway is enhanced. Certainly, when employing the acute tryptophan depletion technique—which lowers Trp availability—in both rodents and humans, there is a decrease in brain 5-HT synthesis. This reduction in 5-HT synthesis can result in a deterioration of mood, particularly in susceptible individuals [18–20]. Moreover, lower circulating levels of Trp caused by inflammation and/or stress have been associated with a reduction in the brain levels of 5-HT and an increase in Kyn downstream metabolites in both unipolar and bipolar depression. For some reviews on this topic please see [10, 21–23]. Nevertheless, certain studies have not identified a relationship between the Kyn pathway and depression [24, 25]. This suggests that the reduced 5-HT activity in the brain may result from heightened catabolism of Trp along the Kyn pathway in a specific subgroup of individuals experiencing depression.

Animal studies have demonstrated that the eCB system is also significantly involved in the pathophysiology and psychopharmacology of MDD [26]. The eCB system is a key neuromodulatory system in the brain and is implicated in synaptic plasticity, development, immune function, response to endogenous and environmental insults, and regulation of stress [27, 28]. The eCB system, including the *N*-arachidonoylethanolamine (AEA) and 2-arachidonoylglycerol (2-AG), primarily acts through two G-protein-coupled receptors, named CB_1_ and CB_2_. The CB_1_ receptor is highly expressed in the brain regions regulating mood, emotion, reward, stress response, and cognition. The CB_2_ receptor modulates immune cell functions and inflammation in the periphery and, in some circumstances, in the brain [29]. Activation of cannabinoid receptors using selective agonists as well as indirectly by inhibiting the enzymes involved in the degradation of AEA (fatty acid amide hydrolase (FAAH)) and/or 2-AG (monoacylglycerol lipase (MAGL)) leads to antidepressant-like activity in several animal models of depression [26, 30]. Importantly, the FAAH inhibitor URB597, which enhances the eCB system, shows antidepressant activity in animal models via an increase of the 5-HT firing activity and 5-HT brain levels [31, 32]. Similarly, the CB1 agonist WIN55,212–2 [33] and delta-9-tetrahydrocannabinol (THC) [34, 35] increase the firing activity of 5-HT neurons. Moreover, both THC and CBD, the two major components of cannabis, reduce the inflammatory cytokine-induced conversion of Trp into Kyn [36]. Although with still limited evidence, human studies have also confirmed the preclinical results highlighting a link between impairments in the eCB system and MDD (for a review see Alcaraz-Silva et al. [37] and Garani et al. [38]). Finally, a significant association of plasma levels of interleukin-6 with 2-AG and picolinic acid (PIC, a downstream metabolite of Kyn) was found in people with psychiatric disorders and controls, but only PIC was associated with neuroticism [39]. A possible association between the two metabolic pathways has also been explored in the pathophysiology and pharmacology of migraine [40].

Currently, no studies in humans have yet examined the possible crosstalk between these two systems and depressive symptoms, which may have important implications for the discovery of novel antidepressant drugs and biomarkers of depression. Therefore, here, we have examined the peripheral biomarkers related to the degradation of Trp into Kyn and 5-HT and the eCB system with the goal of determining if there is a potential crosstalk between these two biochemical systems and the levels of depression in accordance with the Research Domain Criteria (RDoC) approach [41] and understanding if their metabolism is changed according to the depressive condition.

## Methods

### Study participants

Individuals with both unipolar and bipolar depression diagnosed according to the Diagnostic and Statistical Manual of Mental Disorders version 5 (DSM-5) [42] and aged 18–70 years were recruited from the Mood Disorders Program at the McGill University Health Center. They all fulfilled the criteria for a current major depression episode in the last 3 months prior to enrolling in the study. Individuals with comorbid substance use disorder, mood disorder secondary to a medical condition, or currently enrolled in any detoxification program were excluded. Potential subjects were referred by the treating physicians to the principal investigator of the study. All subjects gave a written informed consent to participate in the study after receiving a full explanation of the study protocol, approved by the Ethics Board at the MUHC (number 14–045-PSY) and conducted in accordance with the Declaration of Helsinki and ICH Good Clinical Practice.

A control group composed of healthy individuals defined as the absence of psychiatric disorders according to the Structured Clinical Interview for DMS-5 (SCID-5) [43] and physical diseases was matched for age, sex, gender, and socio-economic status, where possible. They were recruited through public advertisement and by word of mouth among workers at the McGill University Health Center (MUHC).

None of the recruited individuals reported active suicide behavior, assessed as a score < 4 on item 10 of the Montgomery–Åsberg Depression Rating Scale (MADRS) [44] and clinical judgment. Patients were on stable medications for at least 3 months at the time of the psychiatric assessment and blood withdrawal. Patients were taking the following psychoactive medications (alone or in combination): citalopram (20–40 mg/day), escitalopram (10–20 mg/day), fluoxetine (20–60 mg/day), fluvoxetine (40 mg/day), sertraline (25–150 mg/day), duloxetine (120 mg/day), venlafaxine (150–225 mg/day), desvenlafaxine (50 mg/day), bupropione (150–300 mg/day), amitriptyline (10–50 mg/day), desipramine (60 mg/day), mirtazapine (7.5–45 mg/day), trazodone (50–150 mg/day), aripiprazole (1–15 mg/day), lurasidone (20–40 mg/day), olanzapine (2.5–10 mg/day), quetiapine (25–600 mg/day), risperidone (1–2 mg/day), gabapentin (900 mg/day), lamotrigine (75–100 mg/day), lithium (150–1200 mg/day), valproic acid (325–750 mg/day), and methylphenidate (10–40 mg/day). Patients with treatment-resistant depression were defined as having failed at least two adequate trials with different antidepressants in mono or combination therapy at the adequate dose and for at least 3 weeks [45] and were not excluded.

Pregnant women were excluded (positive blood β-HCG test).

The determination of the required study sample size was guided by our prior research [46]. In this earlier study, we identified a correlation between the Kyn/Trp ratio and levels of depressive symptoms measured using the Beck Depression Inventory scale [47]. The effect size (Cohen’s *d*) observed was 0.332. A power analysis was conducted using the G*Power software version 3. The aim was to achieve a comparable effect size with a significance level alpha of 0.05 and a power of 0.8. The analysis revealed that a minimum of 80 individuals was required to attain the desired outcome.

A blood withdrawal was performed between 8 and 10 am after an overnight fast. The blood was left for 30 min to clot at 4 °C protected from light and then centrifuged at 1300 rpm for 10 min at 4 °C. The collected serum was then divided into 200 µl aliquots and stored at − 80 °C until analyses.

### Psychiatric assessment

The presence and severity of depressive symptoms in unipolar, bipolar, and healthy individuals was evaluated by two psychiatrists (GG, NAN) and one physician (MG) with the following behavioral scales: the Montgomery–Åsberg Depression Rating Scale (MADRS) [44], the Clinical Global Impression-Severity of Illness (CGI-S) [48], and the Beck Depression Inventory—Second Edition (BDI-II) [49].

### Determination of serum concentrations of tryptophan, serotonin, and kynurenine

Serum concentrations of Trp, 5-HT, and Kyn were determined using a standard procedure in the lab [14, 50]. Briefly, the compounds were analyzed using a high-performance liquid chromatography system equipped with a Shimadzu RF-10 AXL fluorometric detector (excitation wavelength, 285 nm; emission wavelength, 345 nm) for the determination of Trp and 5-HT, and a Varian ProStar 310 UV–Vis detector set at 360 nm for the quantification of Kyn. The chromatographic separation was performed using an analytical Synergi Fusion-RP 80 A column (4 µm; 250 × 4.6 mm; Phenomenex, Aschaffenburg, Germany) and an isocratic gradient of the mobile phase composed of 8% acetonitrile and 92% phosphate buffer 0.004 M, pH 3.5.

### Determination of serum concentrations of eCBs and related lipids

Serum concentrations of the eCBs, AEA, and 2-AG and three additional lipids that are close in structure to the eCBs (2-oleoylglycerol (2-OG), *N*-oleoylethanolamine (OEA), and palmitoylethanolamide (PEA)) were determined in lipid extracts of serum following previously reported methods [51, 52].

Serum samples (0.4 mL each) were thawed and made up to 15% ethanol to which internal standards [^2^H_8_]-AEA (16.9 pmol) and [^2^H_8_]-2-AG (46.5 pmol) (Cayman Chemicals, Ann Arbor, MI) were added. Samples were vortexed and centrifuged at 12,000 rpm for 4 min. The resulting supernatant was loaded onto Bond Elut C18 solid-phase extraction columns (1 mL: Varian Inc, Lake Forest, CA), previously conditioned with 1 mL redistilled ethanol and 3 mL of double distilled water (ddH_2_O). The remaining pellet was rinsed with 100 µL of 15% ethanol and recentrifuged at 12,000 rpm for 3 min. The resulting supernatant was also loaded onto the C18 column. Columns were washed with 5 mL ddH_2_O (eluate discarded) followed by 1 mL of ethyl acetate (eluate collected). The ethyl acetate layer in the resulting eluate was removed and dried under N_2_. The samples were resuspended twice in ethyl acetate and dried. The final samples were resuspended in 30 µL of methanol and stored at − 80 °C.

Following preparation, the concentrations of eCBs (AEA and 2-AG), along with the related biogenic lipids (PEA, OEA, 2-OG), were quantified in 5 µl of the methanol extract using stable isotope-dilution and electrospray ionization liquid chromatography/mass spectrometry using an Agilent triple quadrupole 6460 mass spectrometer. The chromatography was done using a Phenomenex Luna 5µ C18 250 × 2.0 mm column. The mobile phases were 0.005% v/v acetic acid and 1 mM ammonium acetate in water (A) and in methanol (B). The sequence was 15% A/85% B from 0 to 10 min, 100% B from 10 to 16 min, and 15% A/85% B from 16 to 21 min. All solvents were HPLC grade. Selective ion monitoring, made in the positive ion mode, was used to detect the daughter ions of [^2^H_8_]AEA (356.3 → 62), AEA (348.3 → 62), OEA (326.3 → 62), PEA (300.3 → 62), [^2^H_8_]2-AG and 1(3)-AG (387.4 → 293.1), 2-AG and 1(3)-AG (379.3 → 287.1), and 2-OG and 1(3)-OG (357.3 → 286.1). Standard curves were generated for 2-AG (10–4250 pg/µL), 2-OG (20–8500 pg/µL), AEA (0.2–85 pg/µL), OEA (0.3–127.5 pg/µL), and PEA (1.2–510 pg/µL), each sample containing the internal. Concentrations of the analytes in the samples were determined from standard curves of the area ratios (standard/analyte) versus the concentration ratios (standard/analyte); [^2^H_8_]-AEA was used as the standard for AEA, OEA, and PEA while [^2^H_8_]-2-AG was used for 2-AG and 2-OG. Both 2-AG and 2-OG migrate as doublets in both the standards and samples because of the isomerization of 2-acylgylcerol to 1,3-acylglycerol over time. The areas of both peaks were added for the analyses. The area ratios for all analytes in all samples were within their respective standard curves.

### Statistical analysis

Statistical analysis was conducted using the IBM SPSS statistics 27 (Chicago, IL) and JMP® version Pro 16 software for Windows (SAS Institute Inc., Cary, NC, USA). The relationships between the serum biomarkers of the eCB system and Trp into Kyn degradation or between the serum biomarkers and depression severity were determined by Pearson’s partial correlation controlling for age, BMI, and sex. The Benjamini–Hochberg false discovery rate (FDR) control was implemented to correct for multiple comparisons of nine biomarkers. The non-parametric multivariate method of principal component analysis (PCA) was used to assess any relationships among variables, converting data to principal component scores. Each variable was centered and standardized individually, and the principal components were calculated from eigenvalue decomposition of the correlation matrix. Score plot and loading plot graphs were obtained for the first two principal components. Score plot analyses allow for the evaluation of data clustering. Loading plot analyses indicate how variables correlate with one another, considering that a small angle between vectors implies positive correlation, while a large angle suggests a negative correlation between variables and a 90° angle indicates absence of correlation; the length of the vector represents the variance explained. For data presentation, the biplot was selected in order to show overlay of both score plot and loading plot [53]. Demographic and clinical data were reported as mean ± SD and compared between individuals with the absence or presence of mild/severe depressive symptoms using Student *t*-test for continuous variables and Pearson’s chi-square test for discrete variables. Serum levels of biomarkers were reported as mean ± SD. Differences between individuals without depressive symptoms and individuals with mild/severe depressive symptoms for the serum levels of biomarkers were computed using multivariate analysis of covariance (MANCOVA) with Pillai’s criterion as the significance test and age, BMI, sex, and treatment with a drug enhancing 5-HT levels (selective serotonin reuptake inhibitor (SSRI), selective serotonin norepinephrine reuptake inhibitor (SNRI), tricyclic antidepressant (TCA)) as covariates. MANCOVA with age, BMI, and sex as covariates was also used to test for possible differences in the biomarkers between individuals with mild/severe depressive symptoms in therapy with a drug enhancing 5-HT levels (SSRI, SNRI, or TCA) and individuals with mild/severe depressive symptoms in therapy with a drug not primarily enhancing 5-HT levels (antipsychotic and/or mood stabilizer). Due to multiple testing, Bonferroni correction was applied. Prior to computing statistical analyses, we controlled for possible outliers considering a measure an outlier if it fell out of the 2.2 interquartile range (IQR) [54]. No outliers were detected.

## Results

### Characteristics of the study population

A total of 82 individuals, of whom 35 were diagnosed with unipolar depression, 16 with bipolar depression, and 31 with no psychiatric disorders according to the DSM-5, were recruited. The study population was composed of 41 women and 41 men, and the mean age was 38.7 ± 14.6 years (mean ± SD). The mean age of the first psychiatric diagnosis was 30.6 ± 13.6 years (min 5 and max 66 years), and the mean duration of illness was 12.2 ± 11.4 years (min 1 and max 45 years). Fourteen individuals declared smoking tobacco, and 11 reported having used cannabis in the past.

### 2-AG, AEA, PEA, and Kyn/Trp ratio are positively associated with MADRS, while Trp and 5-HT are negatively associated with MADRS

Table 1 reports the Pearson’s partial correlations between serum concentrations of the eCBs, related lipids, Trp, Kyn, and 5-HT and depressive mood measured with the MADRS, CGI-Severity of illness, and BDI-II scores. Correcting for the possible confounding factors sex, age and BMI, and adjusting for the multiplicity of tests using the FDR control procedure, we found that the serum concentrations of 2-AG, AEA, PEA, Kyn, and the ratio Kyn/Trp were positively associated with the MADRS score, whereas the levels of Trp and 5-HT were negatively associated with the MADRS score. In addition, the total CGI-Severity of illness was positively associated with the serum concentrations of PEA, Kyn, and Kyn/Trp and negatively associated with the concentrations of Trp and 5-HT. Finally, the BDI-II score was positively associated with the ratio Kyn/Trp and negatively associated with the serum concentrations of Trp.

**Table 1:** Correlation of serum lipid biomarkers and tryptophan metabolites with the severity of depression (MADRS, CGI, and BDI-II total scores)

Biomarker	Total MADRS	Total CGI-severity of illness	Total BDI-II
2-AG (pmol/mL)	0.261*	0.124	0.219
2-OG (pmol/mL)	− 0.087	− 0.120	− 0.085
AEA (pmol/mL)	0.263*	0.178	0.211
OEA (pmol/mL)	0.059	0.490	0.014
PEA (pmol/mL)	0.287*	0.230*	0.162
Trp (µg/mL)	− 0.296**	− 0.305**	− 0.260*
Kyn (ng/mL)	0.204	0.277*	0.144
Kyn/Trp*1000	0.299**	0.376**	0.230*
5-HT (ng/mL)	− 0.299**	− 0.251*	− 0.159

Pearson’s partial correlation scores are corrected for sex, age, and BMI. Boldface indicates a significant difference at *alpha level = 0.05 and **alpha level = 0.01 after the false discovery rate (FDR) control procedure

*2-AG* 2-Arachidonoylglycerol, *2-OG* 2-oleoylglycerol, *AEA* anandamide, *OEA* oleoylethanolamide, *PEA* palmitoylethanolamide, *Kyn* kynurenine, *Trp* tryptophan, *5-HT* serotonin, *MADRS* Montgomery Asberg Depression Rating Scale, *CGI* Clinical Global Impression, *BDI-II* Beck Depression Inventory version-II

### Kyn and Kyn/Trp ratio were positively associated with the concentrations of 2-AG, AEA, OEA, and PEA

**Fig. 1:**
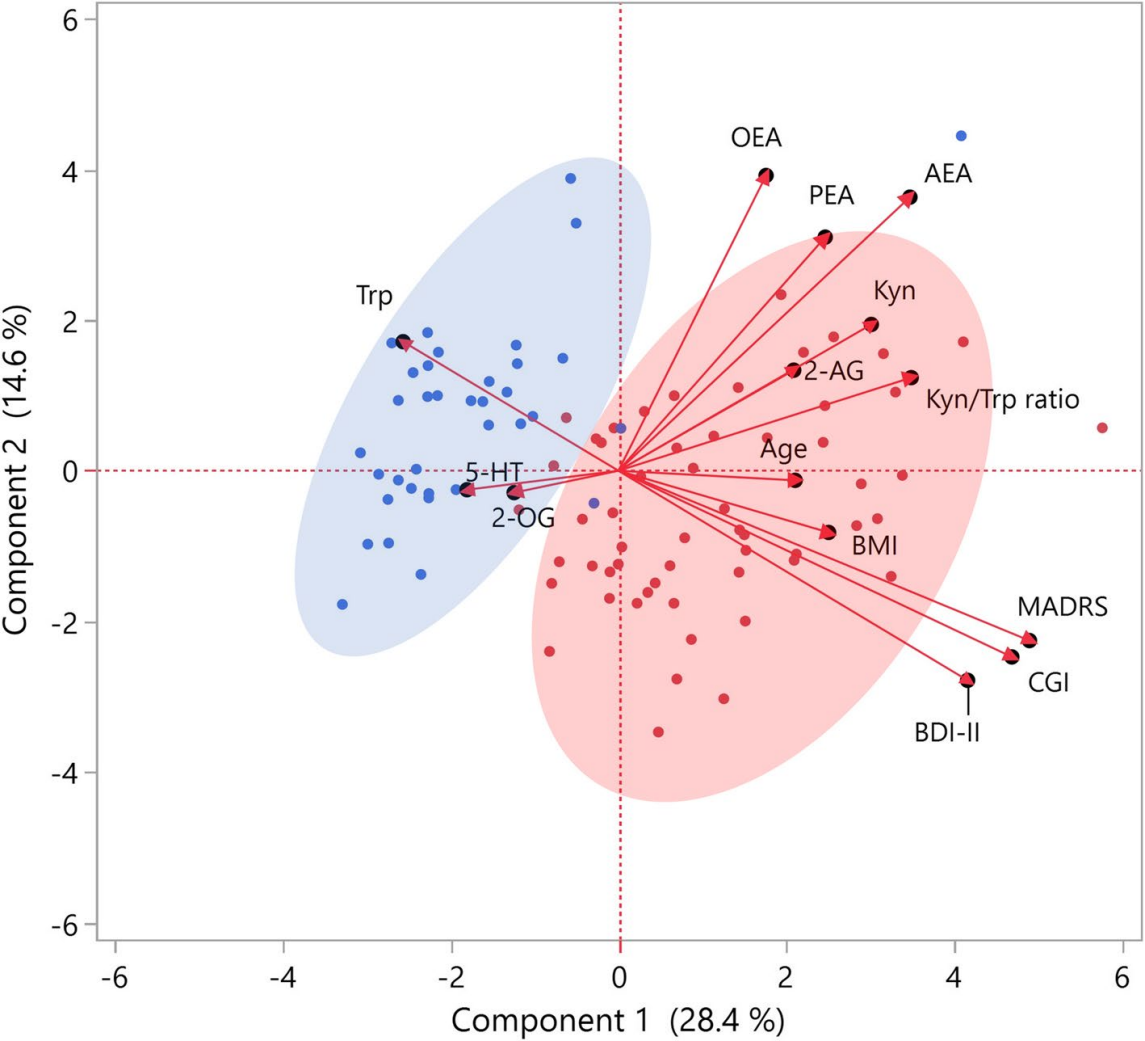
Principal component analysis: the graph is the biplot representing both the score plot and the loading plot. Blue points represent the control group, and red points represent the depression group. Arrows represent the correlation between the parameters and the principal component

We then performed a PCA (Fig. 1), and the specific associations between variables, deduced from the angle defined by the vectors, indicated that Trp, and to a lesser extent 5-HT and 2-OG, were inversely correlated with the MADRS, BDI-II, and CGI-Severity of illness scales, while the Kyn/Trp ratio and Kyn were positively associated with the concentrations of 2-AG, AEA, OEA, and PEA and depressive scales. Remarkably, we observed that these parameters allowed a substantial separation between individuals with absence (blue dots) or presence of mild/severe (red dots) depressive symptoms as shown by the biplot (Fig. 1), although with some overlapping. Upon assessing the contribution of individual variables to the overall classification, it became apparent that the key parameters were BDI-II, CGI-Severity of illness, MADRS, AEA, and OEA, as indicated by their significant vector lengths. Consequently, we chose to explore the interplay of biomarkers both within and between these distinct, well-defined subpopulations.

### Demographics and clinical characteristics of individuals with absence of (MADRS score ≤ 6) and with presence of mild/severe depressive symptoms (MADRS score > 6)

As shown in Table 2, individuals without depressive symptoms were younger, had lower BMI, and MADRS, CGI-severity of illness, and BDI-II scores, as well as lower prevalence of anxiety and personality disorders than those with mild to severe depressive symptoms. There were two patients in remission (MADRS score ≤ 6), one with unipolar and one with bipolar depression, and they were included in the group of asymptomatics. The group with mild/severe depression, compared to the group without symptoms, showed a significant presence of prescription of different class of antidepressant/mood stabilizers pharmacotherapies, and a higher rate of disability or sick leave. Finally, no difference was present between the two groups for marital status.

**Table 2:** Demographic and clinical characteristics of individuals with absence (MADRS score ≤ 6) or mild to severe (MADRS score > 6) depressive symptoms according to the MADRS scale

	Individuals with absence of depressive symptoms (*n* = 33)	Individuals with mild to severe depressive symptoms (*n* = 49)	Statistics
Age, years	33.9 ± 13.8	41.7 ± 14.5	*t* = 2.406; df = 80; *p* = 0.018
Sex, m/f (%)	17 f (52) 16 m (48)	25 f (51) 24 m (49)	*χ*^2^ = 0.00, *p* = 1.00
BMI, kg/m^2^	23.5 ± 3.8	27.9 ± 6.5	*t* = 3.404; df = 80; *p* = 0.001
Total MADRS	2.1 ± 1.9	22.5 ± 8.7	*t* = 12.667; df = 80; *p* < 0.001
Total CGI-severity of illness	1.1 ± 0.4	3.9 ± 1.2	*t* = 12.428; df = 80; *p* < 0.001
Total BDI-II	3.2 ± 3.7	26.2 ± 14.7	*t* = 8.646; df = 80; *p* < 0.001
Diagnosis, *n* (%)			*χ*^2^= 78.05, *p* < 0.001
Unipolar depression	1 (3)	34 (69)^###^	
Bipolar depression	1 (3)	15 (31)^##^	
Pharmacotherapy, *n* (%)			*χ*^2^ = 74.01, *p* < 0.001
Antidepressant	0 (0)	17 (35)^###^	
Antidepressant + antipsychotic	0 (0)	10 (20)^#^	
Antidepressant + antipsychotic + mood stabilizer	0 (0)	12 (25)^###^	
Antidepressant + mood stabilizers	0 (0)	2 (4)	
Antipsychotic + mood stabilizer	2 (6)	8 (16)	
Marital status, *n* (%)			*χ*^2^ = 1.834, *p* = 0.766
Single	19 (58)	29 (59)	
Engaged	1 (3)	1 (2)	
Married	6 (18)	10 (20)	
Common law	4 (12)	3 (6)	
Divorced/separated	3 (9)	6 (13)	
Employment, *n* (%)			*χ*^2^ = 17.813, *p* = 0.001
Unemployed	7 (21)	18 (37)	
Employed	16 (49)	18 (37)	
Disability/sick leave	0 (0)	10 (20)^##^	
Student	9 (27)	2 (4)^##^	
Welfare	1 (3)	1 (2)	
Anxiety disorders, *n* (%)	0 (0)	23 (47)	*χ*^2^ = 20.458, *p* < 0.001
Personality disorders, *n* (%)	1 (3)	24 (49)	*χ*^2^ = 18.541, *p* < 0.001

Comparisons were computed using the Student *T* test for continuous variables and Pearson’s chi-square (*χ*^2^) for categorical variables

*m* males, *f* females, *BMI* body mass index, *MADRS* Montgomery Asberg Depression Rating Scale, *CGI* Clinical Global Impression, *BDI-II* Beck Depression Inventory version-II

^#^*p* < 0.05, ^##^*p* < 0.01, ^###^*p* < 0.001 Pearson’s chi-square

### 2-AG, Kyn, and the ratio Kyn/Trp are higher in individuals with mild/severe depressive symptoms (MADRS score > 6) compared to those asymptomatic (MADRS score ≤ 6), while Trp and 5-HT are lower

Considering the potential influence of age, sex, BMI, and the use of drugs that increase 5-HT levels (TCA, SNRI, or SSRI), a MANCOVA analysis revealed a significant overall difference between individuals with and without mild/severe depressive symptoms (Pillai’s Trace = 0.299; F9,68 = 3.227, *p* = 0.003; partial eta squared = 0.299). This analysis used serum concentrations of the eCBs and related lipids, Trp, Kyn, 5-HT, and the Kyn/Trp ratio as dependent variables. As detailed in Table 3, individuals with mild/severe depressive symptoms exhibited higher serum concentrations of 2-AG and a higher Kyn/Trp ratio, along with lower serum concentrations of Trp compared to those without depressive symptoms. To study for a possible influence of the different pharmacological classes of drugs used by the patients with depressive symptoms on the levels of the eCBs and related lipids and the Trp to Kyn biomarkers, in the group of patients displaying mild/severe depressive symptoms (*n* = 49), we performed a MANCOVA analysis with age, sex, and BMI as possible confounding factors (Pillai’s trace = 0.095; F9,36 = 0.419, *p* = 0.916; partial eta squared = 0.095) comparing those individuals taking a medication impacting 5-HT levels (*n* = 38) and those not taking a drug primarily acting on 5-HT levels (*n* = 11). As shown in Additional file 1: Table S1, no differences in the eCBs and Trp to Kyn biomarkers were observed.

**Table 3:** Comparison of serum concentrations of lipid biomarkers and tryptophan metabolites in individuals with the absence (MADRS score ≤ 6) of and with the presence (MADRS score > 6) of mild to severe depressive symptoms

	Individuals with absence of depressive symptoms (*n* = 33)	Individuals with mild to severe depressive symptoms (*n* = 49)	*F*	Sig	Partial eta squared
2-AG (pmol/mL)	23.28 ± 16.71 (17.26–29.30)	53.00 ± 75.58 (31.52–74.48)	4.560	**0.036**	0.057
2-OG (pmol/mL)	500.3 ± 395.1 (357.9–642.8)	402.3 ± 398.2 (289.1–515.4)	2.302	0.133	0.029
AEA (pmol/mL)	1.52 ± 0.62 (1.30–1.74)	1.79 ± 0.68 (1.60–1.99)	0.332	0.566	0.004
OEA (pmol/mL)	8.33 ± 4.28 (6.78–9.87)	8.12 ± 2.95 (7.27–8.96)	1.587	0.212	0.020
PEA (pmol/mL)	6.80 ± 2.83 (5.78–7.82)	7.18 ± 2.93 (6.34–8.01)	0.027	0.869	0.000
Trp (µg/mL)	13.60 ± 2.64 (12.64–14.55)	11.17 ± 2.14 (10.56–11.77)	9.791	**0.002**	0.114
Kyn (ng/mL)	226.7 ± 110.6 (186.8–266.6)	292.7 ± 84.5 (268.6–316.6)	1.668	0.201	0.021
Kyn/Trp*1000	17.22 ± 8.96 (13.98–20.45)	27.30 ± 10.16 (24.41–30.19)	5.577	**0.021**	0.068
5-HT (ng/mL)	276.2 ± 155.9 (220.0–332.4)	190.9 ± 144.6 (149.7–231.9)	2.495	0.118	0.032

Data are reported as mean ± SD and 95% confidence interval for mean (lower and upper bounds). Multivariate analysis of covariance corrected for sex, age, BMI, and use of a medication affecting 5-HT levels (SSRI, SNRI, or TCA), plus Bonferroni correction for multiple comparisons. Boldface indicates a significant difference at an alpha level = 0.05

*2-AG* 2-arachidonoylglycerol, *2-OG* 2-oleoylglycerol, *AEA* anandamide, *OEA* oleoylethanolamide, *PEA* palmitoylethanolamide, *Kyn* kynurenine, *Trp* tryptophan, *5-HT* serotonin

### *Correlation between serum levels of the eCBs, Trp, Kyn, Kyn/Trp ratio, and 5-HT in individuals without depressive symptoms (MADRS score* ≤ *6)*

**Fig. 2:**
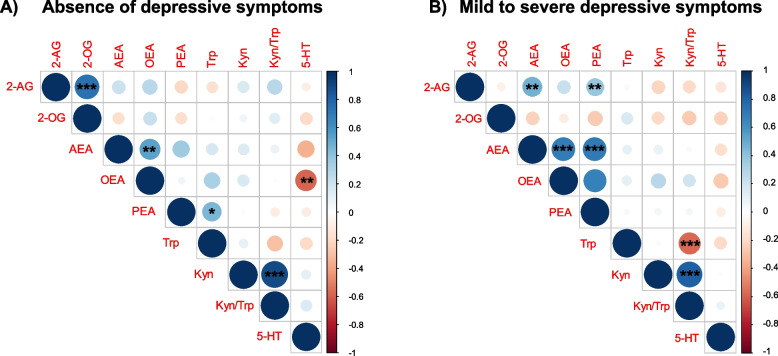
Correlations between eCBs and Trp to Kyn biomarkers in individuals with **A** the absence of depressive symptoms (MADRS score ≤ 6) and **B** the presence of mild to severe depressive symptoms (MADRS score > 6). Correlation scores are corrected for sex, age, and BMI. The legend on the right displays the color-coding of the correlations’ *r* values, with blue colors indicating a positive correlation (*r* > 0) and red colors indicating a negative correlation (*r* < 0). Asterisks within the circles represent significant correlations at *alpha level = 0.05, **alpha level = 0.01, and ***alpha level = 0.001 after the false discovery rate (FDR) control procedure

In individuals without depressive symptoms (Fig. 2A and Additional file 1: Table S2), correcting for sex, age, and BMI, we observed positive and significant associations between serum concentrations of 2-AG and 2-OG, AEA and OEA, Trp and PEA, and Kyn and Kyn/Trp ratio. In contrast, a significant negative association was found between 5-HT and OEA.

### *Correlation between serum levels of the eCBs, Trp, Kyn, Kyn/Trp ratio, and 5-HT in individuals with depressive symptoms (MADRS score* > *6)*

In individuals with depressive symptoms (Fig. 2B and Additional file 1: Table S3), correcting for sex, age, and BMI, we observed a positive and significant association between serum concentrations of 2-AG and AEA, and AEA and OEA. Moreover, PEA was correlated with AEA, 2-AG, and OEA. In contrast, a significant negative correlation was present between Trp and the Kyn/Trp ratio.

## Discussion

In this exploratory work, we investigated the associations among circulating concentrations of the eCBs and their structurally related compounds, the degradation of Trp into 5-HT and Kyn and severity of depression, and found that serum levels of 2-AG, AEA, PEA, Kyn, and the Kyn/Trp ratio were positively associated with the severity of depression, whereas Trp and 5-HT levels were negatively associated with the severity of depression. A principal component analysis revealed a direct correlation of the eCBs with the Kyn/Trp ratio aligning with our hypothesis of a link between these two systems in depression, as indicated by preclinical evidence [26]. Moreover, the results suggest that our study population could be separated in two nearly distinct subgroups characterized by the absence vs presence of mild to severe depressive symptoms according to the MADRS score. We observed higher serum levels of 2-AG and the ratio Kyn/Trp paralleled by lower serum levels of Trp in mild/severe depressive symptoms compared to the asymptomatic group. These data in humans, for the first time, suggest the hypothesis that the Kyn pathway could be the link between eCBs, inflammation, and/or stress and depression [55]. The increased metabolism of Trp into Kyn leading to lower circulating levels of Trp in those individuals with depressive symptoms replicates the results found by our group in different cohorts of depressed patients [13, 14, 50, 56] as well as by other authors [12, 57, 58].

In a study conducted in women with depression, Hill et al. [59] found that depressed women had lower serum 2-AG levels than healthy control females, and this decrease was higher in those depressed females with longer depressive episodes. Interestingly, AEA levels were not associated with depression but negatively correlated with the Hamilton Scale items for cognitive and somatic anxiety. However, in patients with traumatic injury, high concentrations of 2-AG after trauma were correlated with higher depression severity six months later [60]. In our study, individuals with mild/severe depressive symptoms showed higher levels of 2-AG than those with absence of depressive symptoms. Although we controlled for a possible effect of age, sex, BMI, and treatment with antidepressant drugs enhancing 5-HT levels such as SSRIs, SNRIs, or TCAs, we cannot rule out a confounding role played by the pharmacotherapy, especially with psychoactive drugs acting with different mechanisms of action than SSRIs, SNRIs, and TCAs. The sample size of our study was in fact not calculated for this purpose, and more studies are required to include the impact of different pharmacological therapies [61, 62] or electroconvulsive therapy [63], which are known to influence the levels of circulating eCBs but also of Trp to Kyn biomarkers [64, 65].

One of the limitations inherent in our study is the absence of control for potential influences from comorbidities and recent cannabis use, which could have been addressed through urine screening. Since in individuals with mild/severe depressive symptoms we found increased 2-AG levels but also increased levels of the Kyn/Trp ratio, our hypothesis suggests that in depression, there is an elevation in 2-AG levels as a response to heightened inflammation and/or stress.

Recent studies have indeed proposed that the eCB system is activated during episodes of inflammation. Inflammatory cytokines such as INF-gamma, IL-1, IL-6, and TNF-alpha are known to stimulate the IDO enzyme, resulting in an increased degradation of Trp into Kyn, which is measured by a higher Kyn/Trp ratio. This reduced amount of Trp can negatively impact the 5-HT pathway. This theory aligns with the “inflammatory hypothesis” illustrated in the PCA in Fig. 1, showing an inverse correlation between OEA, AEA, PEA, 2-AG, and 5-HT, while these markers are closely associated with Kyn and the Kyn/Trp ratio. These findings support the notion that both the eCB system and the Kyn pathway are both activated in response to stress and inflammation. Stress-induced activation of the HPA axis results in increased cortisol levels, which, in turn, stimulate the TDO enzyme with consequent higher degradation of Trp into Kyn [10], and increase in 2-AG-mediated signaling in several brain regions [66]. Future studies are needed to support these hypotheses by measuring concomitantly circulating inflammatory cytokines and cortisol. Nonetheless, there are additional potential mechanisms connecting these two biological systems. For instance, there is emerging evidence suggesting that brain-derived neurotrophic factor (BDNF) could play a role in modulating Trp metabolism within the Kyn pathway in response to stress [67]. Moreover, the impact of the eCB system on depression may, in part, be attributed to its influence on BDNF and, consequently, the process of neurogenesis [68]. The Trp to Kyn pathway could thus represent a potential mechanism linking the eCB and BDNF systems, providing insight into their involvement in the onset of psychiatric disorders triggered by stress. Although they have different biological targets, the *N*-acylethanolamines (NAEs) AEA, OEA, and PEA have overlapping synthetic and catabolic routes [27]. All are synthesized following activation of the enzyme, *N*‐acyl‐phosphatidyl‐ethanolamine‐selective phospholipase D (NAPE‐PLD), and catabolized by fatty acid amide hydrolase (FAAH). However, unlike AEA and OEA, PEA is a saturated lipid and is also efficiently catabolized in the periphery by *N*-acylethanolamine acid amidase (NAAA) [69]. The absence of correlations between PEA and the other measured NAEs in individuals without depressive symptoms implies that the NAAA pathway plays a more significant role in PEA catabolism compared to FAAH. However, in individuals with mild/severe depressive symptoms, the concentrations of all three NAEs are highly associated, suggesting either that FAAH becomes a more active enzyme or that NAAA a less important regulator of PEA concentrations in depression. Similarly, both 2-AG and 2-OG are catabolized by MAGL, and as expected, their serum concentrations were highly correlated in individuals without depressive symptoms.

In individuals with mild/severe depressive symptoms, the association was no longer evident. These findings warrant further investigation to resolve the mechanistic discord of these lipid-mediators through alternate mechanisms distinct from their conserved MAGL regulation. As a consequence of this imbalance within the eCB system in depression, it is not a surprise that the correlation between PEA and Trp or 5-HT and OEA changes according to the presence or absence of depressive symptoms.

## Conclusions

This exploratory study demonstrates for the first time in humans a plausible association between the eCB system and the Trp to 5-HT and Kyn pathways in the modulation of mood, as emerged by integrated parametric and non-parametric analysis of the data.

In particular, the positive association between Kyn levels, the Kyn/Trp ratio (index of inflammation and stress) and the levels of 2-AG and AEA and PEA could potentially arise as a defensive mechanism designed to mitigate the adverse effects of inflammation and/or stress. Notably, in patients with depressive symptoms, alterations in lipid-derived eCBs were observed, suggesting a lipidome dysfunction in this mental condition.

If substantiated through further validation, these distinctive biochemical patterns have the potential to act as future biomarkers of the severity of MDD and can offer valuable insights for exploring new targets in the MDD psychopharmacology.

### Supplementary Information

**Additional file 1: Table S1.** Comparison of serum concentrations of lipid biomarkers and tryptophan metabolites in individuals with mild to severe depressive symptoms (MADRS score > 6) using a drug acting by increasing 5-HT levels (SSRI, SNRI or TCA) and in those not using a drug primarily acting by increasing 5-HT levels. **Table S2.** Correlation among serum lipid biomarkers and tryptophan metabolites in 33 individuals with absence of depressive symptoms according to a MADRS score ≤ 6. **Table S3.** Correlation among serum lipid biomarkers and tryptophan metabolites in 49 individuals with mild to severe depressive symptoms according to a MADRS score > 6.

## Data Availability

All data associated with this study are available upon reasonable request to the corresponding authors (SC: stefano.comai@unipd.it; GG: gabriella.gobbi@mcgill.ca). A material transfer agreement will be required for the sharing of materials.

## Abbreviations

2-AG: 2-Arachidonoylglycerol
2-OG: 2-Oleoylglycerol
5-HT: Serotonin
AEA: *N*-Arachidonoylethanolamine
BDI-II: Beck Depression Inventory—Second Edition
BDNF: Brain-derived neurotrophic factor
CGI-S: Clinical Global Impression-Severity of Illness
DSM-5: Diagnostic and Statistical Manual of Mental Disorders version 5
eCB: Endocannabinoid
FAAH: Fatty acid amide hydrolase
IDO: Indoleamine 2,3-dioxygenase
IQR: Interquartile range
Kyn: Kynurenine
MADRS: Montgomery-Åsberg Depression Rating Scale
MAGL: Monoacylglycerol lipase
MANCOVA: Multivariate analysis of covariance
MDD: Major depressive disorder
MUHC: McGill University Health Center
NAAA: *N*-Acylethanolamine acid amidase
NAPE‐PLD: *N*‐Acyl‐phosphatidyl‐ethanolamine‐selective phospholipase d
OEA: Oleoylethanolamide
PCA: Principal component analysis
PEA: Palmitoylethanolamide
PIC: Picolinic acid
SCID-5: Structured Clinical Interview for DSM-5
SNRI: Selective serotonin norepinephrine reuptake inhibitor
SSRI: Selective serotonin reuptake inhibitor
TCA: Tricyclic antidepressant
TDO: Tryptophan 2,3-dioxygenase
THC: Delta-9-tetrahydrocannabinol
Trp: Tryptophan
