# Supplementary material for: Dysfunction in endocannabinoids, palmitoylethanolamide, and degradation of tryptophan into kynurenine in individuals with depressive symptoms
Source: BMC Med. 2024 Jan 25;22:33. doi: 10.1186/s12916-024-03248-8 (PMC10809514; doi:10.1186/s12916-024-03248-8)
Supplement: Supplementary file 1 — Additional file 1: Table S1. Comparison of serum concentrations of lipid biomarkers and tryptophan metabolites in individuals with mild to severe depressive symptoms (MADRS score > 6) using a drug acting by increasing 5-HT levels (SSRI, SNRI or TCA) and in those not using a drug primarily acting by increasing 5-HT levels. Table S2. Correlation among serum lipid biomarkers and tryptophan metabolites in 33 individuals with absence of depressive symptoms according to a MADRS score ≤ 6. Table S3. Correlation among serum lipid biomarkers and tryptophan metabolites in 49 individuals with mild to severe depressive symptoms according to a MADRS score > 6. [file 12916_2024_3248_MOESM1_ESM.docx]

**Supplemental Table 1.** Comparison of serum concentrations of lipid biomarkers and tryptophan metabolites in individuals with mild to severe depressive symptoms (MADRS score > 6) using a drug acting by increasing 5-HT levels (SSRI, SNRI or TCA) and in those not using a drug primarily acting by increasing 5-HT levels.

|  | Individuals with depressive symptoms taking SSRI, SNRI or TCA  (n=38) | Individuals with depressive symptoms not taking SSRI, SNRI or TCA  (n=11) | F | Sig. | Partial Eta Squared |
| --- | --- | --- | --- | --- | --- |
| 2-AG  (pmol/mL) | 49.88±74.20 | 68.29±84.10 | 0.722 | 0.400 | 0.016 |
| 2-OG  (pmol/mL) | 443.3±438.5 | 291.9±182.4 | 0.861 | 0.358 | 0.019 |
| AEA  (pmol/mL) | 1.82±0.68 | 1.76±0.69 | 0.446 | 0.508 | 0.010 |
| OEA  (pmol/mL) | 8.39±2.81 | 7.52±3.40 | 0.149 | 0.701 | 0.003 |
| PEA  (pmol/mL) | 7.29±2.75 | 7.03±3.64 | 0.198 | 0.658 | 0.004 |
| Trp  (μg/mL) | 11.14±2.17 | 11.52±1.99 | 0.038 | 0.846 | 0.001 |
| Kyn  (ng/mL) | 299.5±83.1 | 273.4±92.7 | 0.248 | 0.597 | 0.006 |
| Kyn/Trp *1000 | 28.07±10.37 | 24.44±9.82 | 0.149 | 0.702 | 0.003 |
| 5-HT  (ng/mL) | 187.3±145.8 | 186.4±142.3 | 0.057 | 0.812 | 0.001 |

Data are reported as mean ± SD. Multivariate analyses of covariance corrected for sex, age and BMI plus Bonferroni post-hoc correction for multiple comparisons. SSRI: Selective Serotonin Reuptake Inhibitor; TCA: tricyclic antidepressant; MADRS: Montgomery Asberg Depression rating scale; 2-AG: 2-Arachidonoylglycerol; 2-OG: 2-oleoylglycerol; AEA: *N*-arachidonoylethanolamine; OEA: *N-*oleoylethanolamine; PEA: palmitoylethanolamide; Kyn: Kynurenine; Trp: Tryptophan; 5-HT: Serotonin.

**Supplemental Table 2.** Correlation among serum lipid biomarkers and tryptophan metabolites in 33 individuals with absence of depressive symptoms according to a MADRS score ≤ 6.

|  | 2-AG (pmol/mL) | 2-OG (pmol/mL) | AEA (pmol/mL) | OEA (pmol/mL) | PEA (pmol/mL) | Trp (μg/mL) | Kyn (ng/mL) | Kyn/Trp *1000 |
| --- | --- | --- | --- | --- | --- | --- | --- | --- |
| 2-AG (pmol/mL) |  |  |  |  |  |  |  |  |
| 2-OG (pmol/mL) | **0.740***** |  |  |  |  |  |  |  |
| AEA (pmol/mL) | 0.217 | -0.178 |  |  |  |  |  |  |
| OEA (pmol/mL) | 0.274 | 0.225 | **0.539**** |  |  |  |  |  |
| PEA (pmol/mL) | -0.208 | -0.177 | 0.358 | 0.065 |  |  |  |  |
| Trp (μg/mL) | -0.162 | -0.021 | 0.177 | 0.326 | **0. 459*** |  |  |  |
| Kyn (ng/mL) | 0.168 | 0.069 | 0.160 | 0.167 | 0.022 | 0.108 |  |  |
| Kyn/Trp *1000 | 0.277 | 0.139 | 0.088 | -0.019 | -0.103 | -0.296 | **0.897***** |  |
| 5-HT (ng/mL) | -0.093 | -0.195 | -0.358 | **-0.582**** | -0.109 | -0.193 | 0.115 | 0.153 |

Correlation scores are corrected for sex, age and BMI. Boldface indicates significant difference at * alpha level=0.05, ** alpha level=0.01 and *** alpha level=0.001 after false discovery rate (FDR) control procedure. MADRS: Montgomery Asberg Depression rating scale; 2-AG: 2-Arachidonoylglycerol; 2-OG: 2-oleoylglycerol; AEA: Anandamide; OEA: Oleoylethanolamide; PEA: palmitoylethanolamide; Kyn: Kynurenine; Trp: Tryptophan; 5-HT: Serotonin.

**Supplemental Table 3.** Correlation among serum lipid biomarkers and tryptophan metabolites in 49 individuals with mild to severe depressive symptoms according to a MADRS score > 6.

|  | 2-AG (pmol/mL) | 2-OG (pmol/mL) | AEA (pmol/mL) | OEA (pmol/mL) | PEA (pmol/mL) | Tryptophan (μg/mL) | Kynurenine (ng/mL) | Kyn/Trp *1000 |
| --- | --- | --- | --- | --- | --- | --- | --- | --- |
| 2-AG (pmol/mL) |  |  |  |  |  |  |  |  |
| 2-OG (pmol/mL) | -0.085 |  |  |  |  |  |  |  |
| AEA (pmol/mL) | **0.478**** | -0.226 |  |  |  |  |  |  |
| OEA (pmol/mL) | 0.220 | -0.102 | **0.693***** |  |  |  |  |  |
| PEA (pmol/mL) | **0.383**** | -0.259 | **0.708***** | **0.671***** |  |  |  |  |
| Trp (μg/mL) | 0.031 | 0.168 | 0.110 | 0.111 | 0.042 |  |  |  |
| Kyn (ng/mL) | -0.217 | -0.199 | 0.095 | 0.276 | 0.059 | -0.026 |  |  |
| Kyn/Trp *1000 | -0.196 | -0.251 | -0.037 | 0.203 | 0.057 | **-0.575***** | **0.811***** |  |
| 5-HT (ng/mL) | -0.138 | -0.225 | -0.179 | -0.267 | -0.098 | -0.193 | -0.016 | 0.084 |

Correlation scores are corrected for sex, age and BMI. Boldface indicates significant difference at * alpha level=0.05, ** alpha level=0.01 and *** alpha level=0.001 after false discovery rate (FDR) control procedure. MADRS: Montgomery Asberg Depression rating scale; 2-AG: 2-Arachidonoylglycerol; 2-OG: 2-oleoylglycerol; AEA: Anandamide; OEA: Oleoylethanolamide; PEA: palmitoylethanolamide; Kyn: Kynurenine; Trp: Tryptophan; 5-HT: Serotonin.
